# Development and validation of risk score for predicting positive repeat prostate biopsy in patients with a previous negative biopsy in a UK population

**DOI:** 10.1186/1471-2490-9-7

**Published:** 2009-07-16

**Authors:** Mark A Rochester, Nora Pashayan, Fiona Matthews, Andrew Doble, John McLoughlin

**Affiliations:** 1 Department of Urology, Addenbrooke's Hospital, Cambridge, UK; 2 Department of Urology, West Suffolk Hospital, Bury St Edmunds, UK; 3 Institute of Public Health, University of Cambridge School of Clinical Medicine, Cambridge, UK; 4 Medical Research Council Biostatistics Unit, Institute of Public Health, University of Cambridge School of Clinical Medicine, Cambridge, UK

## Abstract

**Background:**

Little evidence is available to determine which patients should undergo repeat biopsy after initial benign extended core biopsy (ECB). Attempts have been made to reduce the frequency of negative repeat biopsies using PSA kinetics, density, free-to-total ratios and Kattan's nomogram, to identify men more likely to harbour cancer but no single tool accurately predicts biopsy outcome. The objective of this study was to develop a predictive nomogram to identify men more likely to have a cancer diagnosed on repeat prostate biopsy.

**Methods:**

Patients with previous benign ECB undergoing repeat biopsy were identified from a database. Association between age, volume, stage, previous histology, PSA kinetics and positive repeat biopsy was analysed. Variables were entered stepwise into logistic regression models. A risk score giving the probability of positive repeat biopsy was estimated. The performance of this score was assessed using receiver characteristic (ROC) analysis.

**Results:**

110 repeat biopsies were performed in this period. Cancer was detected in 31% of repeat biopsies at Hospital (1) and 30% at Hospital (2). The most accurate predictive model combined age, PSA, PSA velocity, free-to-total PSA ratio, prostate volume and digital rectal examination (DRE) findings. The risk model performed well in an independent sample, area under the curve (AUC_ROC_) was 0.818 (95% CI 0.707 to 0.929) for the risk model and 0.696 (95% CI 0.472 to 0.921) for the validation model. It was calculated that using a threshold risk score of > 0.2 to identify high risk individuals would reduce repeat biopsies by 39% while identifying 90% of the men with prostate cancer.

**Conclusion:**

An accurate multi-variable predictive tool to determine the risk of positive repeat prostate biopsy is presented. This can be used by urologists in an outpatient setting to aid decision-making for men with prior benign histology for whom a repeat biopsy is being considered.

## Background

The majority of the 35000 new diagnoses of prostate cancer annually in the UK [1] are made as a result of transrectal ultrasound-guided (TRUS) biopsies of the prostate performed to investigate an elevated serum PSA or abnormal digital rectal examination. Seventy-five percent of cancers identified are detected at the first biopsy [2]. The majority of men undergoing this procedure, however, will not have a cancer detected and doubt will remain both in their minds and in those of the clinician as to whether a further biopsy is required in the setting of a persistently abnormal or rising serum PSA. Prostate biopsy is not without morbidity [3] carrying a 1% risk of sepsis or severe haemorrhage, and 5% risk of urinary tract infection, and the need to reduce unnecessary repeat biopsies was further highlighted recently in the UK with the publication of NICE guidelines for prostate cancer [4]. This document estimated that 89000 prostate biopsies are performed annually in the UK. Twenty percent of cancers are detected at the second biopsy session [5], and a number of parameters have been promoted to reduce the frequency of benign repeat biopsy. Abnormal digital rectal examination (DRE) [6], PSA [5], PSA velocity > 0.75 ng/ml/yr [7], PSA density > 0.15 [5,7] transition zone density > 0.25 [5,8], and free-to-total PSA ratio [5,7], as well as initial histological findings of high grade prostatic intraepithelial neoplasia (HGPIN) [6,8] or suspicious histology [9] have all been used to identify those men at higher risk of positive repeat biopsy. These individual parameters when taken alone, have poor positive and negative predictive value [5]. Kattan has produced a number of predictive nomograms to aid decision making in prostate cancer [10], including one intended to enhance prediction of positive repeat biopsies, based on sextant prostate biopsy data [11]. It was suggested that the model could be improved through adding free-to-total PSA ratio and prostate volume to the calculation. The aim of this study was to use a similar approach for a United Kingdom population in order to produce a more accurate predictive tool to be used in the outpatient setting when faced with a patient who may be a candidate for a second TRUS and biopsy of the prostate.

## Methods

All patients who underwent first repeat prostate biopsy sessions at two hospitals in the former West Anglia Cancer Network with a combined catchment population of more that 1.25 million were studied. Those who had an initial negative biopsy were analysed. Indications for repeat biopsy and the number of biopsy cores taken were similar in both hospitals according to local guidelines, with an extended core biopsy template (at least 10 cores including anterior horn of the peripheral zone). Prostate biopsies were performed using TRUS guidance with a 7.5 MHz probe (B&K), biopsy gun (Bard) and an 18 gauge biopsy needle with local anesthesia.

The clinical parameters recorded for each patient were age, serum PSA, PSA velocity in ng/ml/year (measured over 18 months), prostate volume, DRE findings, time interval between biopsy sessions, and history of HGPIN or suspicious histology. Data was collected from an anonymised database. Research was carried out in compliance with the Helsinki Declaration.

### Developing the risk score

This was based on data obtained from patients undergoing repeat biopsy at Hospital (1) in the former West Anglia Cancer Network during 2007. Using univariate analysis, the association between the study variables (age, prostate volume, DRE findings (clinically benign referred to as stage 1, and a palpable abnormality stage 2), previous suspicious biopsy, previous high grade PIN, PSA, free-to-total PSA, PSA density, PSA velocity) and the outcome of repeat biopsy was studied. These variables were subsequently entered stepwise into logistic regression models. For each model, the mean probability of having a positive repeat biopsy was estimated for each individual. These results were compared with the true status and the coefficients from the model that best discriminated between positive and negative biopsy were used to estimate the risk score. Risk factors that were not statistically significant predictors of positive repeat biopsy were also included into the risk predictor model and to the risk score estimation as this could increase predictive power. Although DRE findings and previous HGPIN did not increase the predictive power of the statistical model, they were included in the risk score calculations as a consequence of their assumed clinical importance.

Probability of positive repeat biopsy was estimated as the expit (*α *+ *β*_1 _*x*_1 _+ *β*_2 _*x*_2 _... β_*n *_*x*_*n *_) of the model where, β represents the coefficient and x the variable used in the risk score: age at biopsy, previous HGPIN, DRE findings, volume of the prostate, serum level of PSA and free-to-total PSA, and PSA velocity. The risk score was calculated for each subject with no missing data points in the selected risk factors.

To assess performance of the risk score with respect to predicting positive repeat biopsy, receiver operating characteristic (ROC) curves were plotted for the risk scores. The area under the curve (AUC) and 95% confidence intervals (CI) were then estimated. A larger area under the ROC curve reflects better performance of a diagnostic test. Sensitivity, specificity, and positive (PPV) and negative predictive values (NPV) and their 95% CI were calculated for various cut-off points of the calculated risk score. The sensitivity of the risk score is the proportion of men with prostate cancer correctly identified as such by the risk score (percentage true positives). The specificity of the risk score is the proportion of men without prostate cancer who are correctly identified as such by the risk score (percentage true negatives). The positive predictive value of the risk score is the proportion of men with a positive result on the risk score who indeed have prostate cancer identified on biopsy.

Analyses were performed using the statistics package Stata 9. (Stata Corp. Stata Statistical Software: release 9. 2005. Texas: Stata Corporation)

### Testing the risk score

The performance of the risk score in predicting prostate cancer diagnosis on repeat biopsy was evaluated in an independent sample of patients who had their repeat prostate biopsy at Hospital (2) in the former West Anglia Cancer Network.

The risk score for each subject was calculated. The scores were tested for sensitivity, specificity, positive predictive value (PPV), and negative predictive value (NPV) in differentiating men with and without prostate cancer diagnosis on repeat biopsy.

## Results

### Developing the risk score -Hospital (1)

A total of 87 patients underwent repeat biopsy at Hospital (1) during the study period, of whom 31% (27) had prostate cancer diagnosis on repeat biopsy. Table 1 shows the association between the outcome of prostate biopsy and the study variables. Only velocity of PSA was statistically significantly higher in men with positive repeat prostate biopsy as compared to men with negative biopsy findings (p = 0.023). Even after adjusting for the other study variables, velocity remained statistically significant predictor of positive repeat biopsy (OR-trend = 1.34; 95%CI 1.03 – 1.74) (Table 2). Though the other variables were not statistically significant predictors individually, they were included in the final logistic regression model as they improved prediction.

**Table 1:** B Repeat prostate biopsy outcome and patient and prostate related factors, Hospital (1) sample

**Variable**	**Prostate cancer detected**			**Prostate cancer not detected**			
	**No of patients**	**Mean (SD)**	**%**	**No of patients**	**Mean (SD)**	**%**	**P-value**

	**N = 27**			**N = 59**			

Mean Age (years)	27	66.7 (7.8)		59	65.2 (7.5)		0.411

Prostate volume (ml)	22	51.1(27.3)		59	61.1 (24.5)		0.097

PSA (ng/ml)	27	12.4 (7.0)		59	11.7 (9.7)		0.735

Free-to-total PSA (%)	24	13.8 (7.4)		56	16.9 (8.4)		0.131

Velocity (ng/ml/year)	21	3.3 (3.2)		43	-0.8 (7.7)		0.023

DRE							0.443

1 (normal)	20		74.1	48		81.4	

2 (abnormal)	7		25.9	11		18.6	

Previous suspicious							0.867

Yes	2		7.4	5		8.5	

No	25		92.6	54		91.5	

Previous HGPIN							0.777

Yes	10		37.0	20		33.9	

No	17		63.0	39		66.1	

SD – standard deviation

P-value derived from logistic regression model

DRE 1 refers to a palpably normal gland, DRE 2 to a abnormal digital rectal examination

**Table 2:** Adjusted β-coefficients and 95% confidence intervals for each risk factor, Hospital (1) sample

**Variable**	**Adjusted β-coefficient**	**95%CI**	
Age (yrs)	0.109	-0.004	0.221

DRE	0.093	-1.536	1.722

Previous HGPIN	-0.120	-1.652	1.412

Volume (ml)	-0.002	-0.030	0.026

PSA (ng/ml)	-0.100	-0.212	0.012

Free-to-total (%)	-0.119	-0.233	-0.005

Velocity (ng/ml/year)	0.293	0.029	0.557

constant	-5.358	-12.389	1.672

* Adjusted for all the other variables in the model

A model that included the variables age, DRE findings, previous HGPIN, volume of the prostate gland, serum levels of PSA and free/total PSA, and PSA velocity had a probability of predicting prostate cancer of 51% compared to 24% in predicting absence of cancer on prostate repeat biopsy.

The coefficients obtained from the logistic regression model are shown in Table 2.

**Figure 1:**
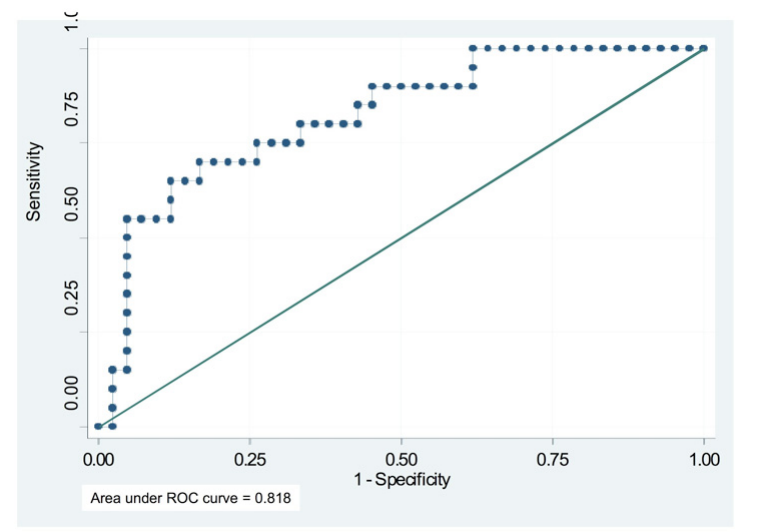
**Receiver operating characteristic curve for the performance of risk score in identifying patients with prostate cancer diagnosis on repeat biopsy, Hospital (1) sample**.

The performance of the risk score in differentiating patients with positive and negative biopsy outcome is shown in the receiver operating characteristic curve in Figure 1, with area under the curve (AUC) = 0.818 (95%CI 0.707 to 0.929).

The risk score cut off that maximised the sum of sensitivity and specificity was derived from ROC analysis. This cut point was 0.45, with sensitivity of 70% and specificity of 83%. A risk score > 0.45, would reduce 69% of the biopsies (43/62) while identifying 65% (13/20) of the cases. Whereas a risk score > 0.2 would reduce 39% (24/62) of the biopsies, while identifying 90% (18/20) of the patients with prostate cancer and missing 10% (2/20) of the patients with prostate cancer. Almost half of the patients with a risk score > 0.2 are likely to have positive prostate biopsy for prostate cancer (PPV = 47.4%; 95% CI 31.0% to 64.2%) (Table 3).

**Table 3:** Sensitivity, specificity, positive predictive value (PPV), negative predictive value (NPV) and 95% confidence intervals (CI) at different risk score cut points, Hospital (1) sample

**Risk score cut point**	**Sensitivity**			**Specificity**			**PPV**			**NPV**		
	
		**95% CI**		**%**	**95% CI**		**%**	**95% CI**		**%**	**95% CI**	
> 0.1	90.0	68.3	98.8	38.1	23.6	54.4	40.9	26.3	56.8	88.9	65.3	98.6

> 0.2	90.0	68.3	98.8	52.4	36.4	68	47.4	31.0	64.2	91.7	73.0	99

> 0.3	75.0	50.9	91.3	71.4	55.4	84.3	55.6	35.3	74.5	85.7	69.7	95.2

> 0.4	70.0	45.7	88.1	83.3	68.6	93.0	66.7	43.0	85.4	85.4	70.8	94.4

> 0.45	65.0	40.8	84.6	85.7	71.5	94.6	68.4	43.4	87.4	83.7	69.3	93.2

> 0.5	55.0	31.5	76.9	88.1	74.4	96.0	68.8	41.3	89.0	80.4	66.1	90.6

> 0.6	35.0	15.4	59.2	95.2	83.8	99.4	77.8	40.0	97.2	75.5	61.7	86.2

> 0.7	10.0	1.2	31.7	97.6	87.4	99.9	66.7	9.4	99.2	69.5	56.1	80.8

> 0.8	0.0	0.0	16.8	97.6	87.4	99.9	0.0	0.0	97.5	67.2	54.0	78.7

### Validation of the risk score

Overall, during the study period, 23 patients had repeat prostate biopsy in Hospital (2), of whom 7 patients (30%) had prostate cancer diagnosis. The characteristics of patients who had repeat biopsy in Hospital (2) are given in Table 4.

**Table 4:** The association between the outcome of prostate biopsy and patient and prostate related factors, Hospital (2) sample

**Variable**	**Prostate cancer detected**			**Prostate cancer not detected**			
	**No of patients**	**Mean (SD)**	**%**	**No of patients**	**Mean (SD)**	**%**	**P-value**

	**N = 7**			**N = 16**			

Age	7	66.6 (5.2)		16	62.8 (6.7)		0.202

Prostate volume	7	35.0 (15.3)		16	49.8 (9.3)		0.025

PSA	7	15.9 (17.7)		16	7.9 (3.0)		0.189

Free/total PSA (%)	7	50.1 (26.3)		15	25.3 (19.9)		0.039

Velocity	7	4.3 (7.8)		16	0.8 (1.9)		0.256

DRE				16			0.457

1 (normal)	7		100.0	15		93.7	

2 (abnormal)	0		0.0	1		6.3	

Previous suspicious							0.907

Yes	1		14.3	2		12.5	

No	6		85.7	14		87.5	

Previous HGPIN							0.095

Yes	0		0.0	5		31.2	

No	7		100.0	11		68.8	

SD – standard deviation

P-value derived from logistic regression model

**Figure 2:**
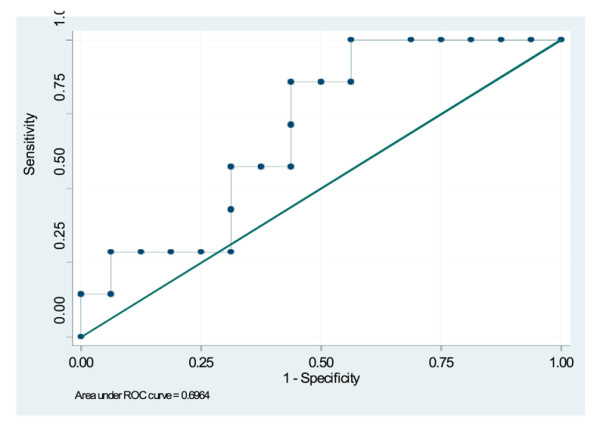
**Receiver operating characteristic curve for the performance of risk score in identifying patients with prostate cancer diagnosis on repeat biopsy, Hospital (2) sample**.

The performance of the risk score in differentiating patients with positive and negative biopsy outcome is shown in the receiver operating characteristic curve in Figure 2, with AUC = 0.696 (95%CI 0.472 to 0.921). This indicates that the risk model has performed well in an independent sample. Using a cut point of 0.2 from the model derived in Hospital (1), 100% (7/7) of patients with positive repeat biopsy were correctly identified.

## Discussion

Clinicians use various triggers for proceeding to repeat prostate biopsies. Serum PSA, (which may be age-adjusted), age, DRE findings, free-to-total PSA ratio, transition zone PSA density, standard PSA density, PSA velocity and the finding of HGPIN on initial biopsy have all been used in this setting [1-5].

The predictive value of these individual factors is limited, however, and this has led to attempts to refine the prediction process and reduce the frequency of unnecessary repeat biopsies. Artificial neural networks and nomograms have been used to this end [11-14] but have not gained wide acceptance in clinical practice.

The detection rate of cancer on second and further biopsies is thought to be lower than the initial procedure. Kattan's group showed that the rate decreased from 19.5% in the second biopsy to 13.5% after 5 or more biopsy sessions [11]. This data and previous studies were, however, based on initial sextant biopsies. In our study, the rate of positive biopsies remained steady at 30%, and the distribution of cancer grade did not change suggesting that even in the era of extended core prostate biopsies initial benign biopsies do not preclude a diagnosis of significant cancer on subsequent investigation.

Identifying those men who require repeat biopsy whilst avoiding unnecessary biopsies is not straightforward. Djavan found that free-to-total PSA ratio and transition zone PSA density were useful predictive factors with AUCs of 74% and 69% [13]. Keetch et al have shown PSA density and PSA velocity to be better predictors [15] suggesting that using more than one variable in determining further biopsy is beneficial, but that the ideal combination is unknown.

The Kattan nomogram included a selection of risk factors that can predict the presence of cancer, and this model was validated in a second repeat biopsy population with an AUC of 71% [14].

Gallo [16] and Akhavan [17] have recently questioned the predictive value of HGPIN in repeat biopsy, and our model supports this view, as adding this variable to the regression model did not increase the accuracy of prediction. However, other authors have suggested that the finding of multifocal HGPIN should be viewed with more suspicion [18].

Kattan showed that incorporating multiple variables can vastly improve on the predicted probability of having prostate cancer [11]. We have adapted this approach to the extended core repeat prostate biopsy population in a UK setting, and improved the accuracy through adding the additional variables of prostate volume and free-to-total PSA ratio.

This nomogram (AUC 0.82, 95%CI 0.71 – 0.93) performed favourably when compared with Kattan's which had an AUC of 0.75. This may be a result of the incorporation of additional variables to the model. This predictive tool is based on men who present either with symptoms, or as a result of opportunistic screening. Whether this predictive tool is applicable to a setting where PSA testing for screening purposes is more widely available needs to be investigated. This model has been internally validated, but re-validation on external populations should be the next step.

The decision to repeat a prostate biopsy must be made after informed discussion between clinician and patient, following a multi-disciplinary team meeting case discussion. A nomogram can aid decision making in this setting. The probability of cancer on repeat biopsy generated by this predictive tool reflects the probability that a man with the same clinical parameters investigated in the same manner as those included in this retrospective analysis would have a positive repeat biopsy. This figure will be interpreted by the patient according to his perception of risk to inform his choice. A precise cut-off to trigger a repeat biopsy is difficult to produce, and will vary according to an individual patient's and urologist's perception of risk, however, using a calculated risk score threshold for re-biopsy of 0.45 (sensitivity 70%, specificity 83%) would reduce the number of unnecessary biopsies in this study (absolute reduction 69%) while identifying 65% of the cancers. Alternatively a more conservative approach using a risk-score threshold for biopsy of > 0.2 would reduce the number of biopsies by 39% while identifying 90% of patients with cancer. As such this study has policy implications. Implementing such a threshold risk score to trigger repeat biopsy could reduce the number of repeat biopsies in the UK by up to 4100 per year, equating to a saving of €1.25 million, prevention of 40 life-threatening hospital admissions for urinary sepsis, and 200 fewer urinary tract infections.

### Strengths of the study

**Figure 3:**
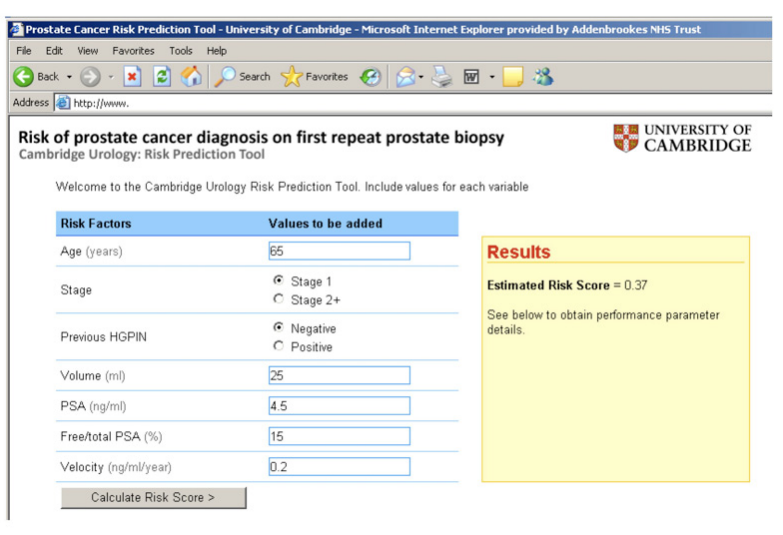
**Example screenshot of Webtool **(available at ).

The predictive tool was developed based on data from one population sample and was tested in an independent sample. Application of the risk scores to an independent sample with good and comparable performance supports the validity of the developed risk score model. A webtool has been developed which is accessible, easy to use, and is based on parameters readily available to the clinician during an outpatient consultation. This facilitates discussion of the risk estimates in real time with the patient and further management planned in an informed fashion (Figure 3).

### Limitations

The risk prediction model and the validation are based on a small sample size. Nevertheless, the model had high predictive accuracy of 82% and has been validated on a small external sample. This is a pilot model pending further work on a larger sample size from different regions of the UK. Repeat prostate biopsy is clearly not performed on all patients and as such a true sensitivity and specificity for the test are impossible to determine. Patients were filtered through clinical judgement. However, as the data are derived over a short period of time, then clinical decision and investigation are likely to be comparable among the patients undertaking repeat biopsy.

This tool is not designed to replace clinical judgement. Once biopsy is considered, the tool will assist in informed decision making.

## Conclusion

We have developed a web-based predictive tool for a UK population which is comparable to previous published predictive nomograms to aid decision-making in men with persistently abnormal PSA who are considering a repeat prostate biopsy. Using such risk score prediction model in clinical practice, could help in forming a strategy for reducing the number of unnecessary repeat biopsies.

## Competing interests

The authors declare that they have no competing interests.

## Authors' contributions

MR participated in design of the study, collected data and drafted the manuscript. NP and FM carried out the statistical analysis and created the predictive model and participated in design of the study. AD participated in the design of the study, collection of data and the drafting of the manuscript. JM conceived of the study, collected data, and participated in study design and coordination. All authors read and approved the final manuscript.

## Pre-publication history

The pre-publication history for this paper can be accessed here:


